# Latent Profile Analysis of Self-Supporting Ability among Rural Empty-Nesters in Northwestern China

**DOI:** 10.3390/ijerph20010711

**Published:** 2022-12-30

**Authors:** Lanzhi Wei, Jianou Xu, Caifeng Luo, Rongzhu Lu, Hui Shi

**Affiliations:** Department of Medical College, Jiangsu University, Zhenjiang 212000, China

**Keywords:** self-supporting ability, sense of coherence, rural empty-nesters, latent profile analysis, northwestern China

## Abstract

The present study aimed to examine the multi-faceted self-supporting ability profiles of rural empty-nesters in northwestern China on the basis of the self-care ability, economic self-support ability, health self-maintenance ability, physical health self-maintenance ability, and psychological health self-maintenance ability using latent profile analysis. It identified the association of self-supporting ability profiles with demographic variables and sense of coherence. The analysis included 1066 participants (mean age = 70.2; SD = 4.3). The results of latent profile analysis identified three distinctive patterns of self-supporting ability―low physical health self-maintenance ability (C_1_, 20.5%), low psychological health self-maintenance ability (C_2_, 31.4%), and high social self-adaption ability (C_3_, 48.0%). The specific demographic variable age (*p* < 0.05), monthly income (*p* < 0.05), education level (*p* < 0.05), how often their children visit (*p* < 0.05), how often their children contact them (*p* < 0.05), whether they drink (*p* < 0.05), the frequency of physical exercise (*p* < 0.05), relationship with children (*p* < 0.05), relationship with neighbours (*p* < 0.05), medical insurance (*p* < 0.05), and the number of chronic diseases (*p* < 0.05) were significantly different among the identified three profiles. A statistically significant positive association existed between self-supporting ability profiles and sense of coherence (SOC) (*p* < 0.001). The results of multinomial logistic regression showed that a greater sense of coherence (SOC), age ≥ 80, monthly income (RMB) (RMB is the abbreviation for Renminbi) < 1000, a good relationship with neighbours, and one type of chronic disease were significantly associated with C_1_ when compared with C_3_ (*p* < 0.05). Furthermore, a greater SOC, their children visiting and contacting them many times per week or once per week were more significantly related to C_2_ than to C_3_ (*p* < 0.05). This study revealed three groups of self-supporting ability and its related predictors in empty-nesters. The predictors related to particular classes of self-supporting ability can provide information for targeted interventions to improve the self-supporting ability of empty-nesters living in rural areas.

## 1. Introduction

The ageing of the natural population is a global phenomenon [1]. There are 260 million individuals aged 60 years or older in China, constituting 18.7% of the total population [2]. The degree of ageing in rural areas is significantly higher than that in cities and towns, which may result from China’s overall economic and social environment, such as the accelerated process of urbanization and the flow of surplus rural labour to large and eastern coastal cities [3]. One of the adverse effects of the ageing population is the increase in the number of empty-nesters in rural areas who do not have children or whose children have left home, leaving them to live alone or with their spouses [1]. The population of empty-nesters in China has exceeded 100 million, with nearly 50% living in rural areas [4]. Rural empty-nesters, as a specific group, lack the life companionship and emotional comfort of adult children during the bidirectional transition between the life cycle and family cycle [3]. In the traditional Chinese family pattern, when parents cannot take care of themselves, they live with their children and receive assistance. In recent years, however, the disintegration of the extended family has meant that often no grown-up children are available to help older adults when required [3]. As age increases, the physiological function of the empty-nesters deteriorates. In addition, they need to strive to deal with psychosocial problems, including loneliness, anxiety disorders, and depression [3]. Meanwhile, the relatively underdeveloped infrastructure and medical conditions in rural areas in northwestern China also result in a low availability of social resources, particularly for those with physical and psychological problems [1]. Furthermore, the phenomenon of “empty nesting” has weakened the function of family protection in rural areas, impacted the social foundation of “raising children to provide against old age and family pension”, and subverted the traditional filial ethic that “children should not travel far when their parents are here” [5]. The factors mentioned above jointly lead to increasingly serious problems for older adults receiving care. Therefore, the problem of providing care for empty-nesters in rural areas has become a social issue that urgently needs to be solved.

It is very common in rural China for older adults to obtain support primarily on their own [6]. The experience of rural empty-nesters in self-supporting requires a direction based on Orem’s Self-Care Theory and active ageing. According to Orem’s Self-Care Theory, individuals usually have three main areas of requisites. They have general self-care requisites, such as eating, dressing, and undressing, as well as controlling bowel and urine and so on; self-care requisites related to growth and development, such as socialising with others and being cared for by family members and so on; and health-related self-care requisites, such as maintaining physical and mental health [7]. Furthermore, Orem’s Self-Care Deficit Theory is structured in guiding subsystems for a systematic nursing practice, such as the Self-Care Theory, the Nursing Systems Theory, and the Self-Care Deficit Theory [8]. Active ageing is applicable both at the individual and population level, aimed at allowing people to accomplish their full potential in physical, social, and mental well-being throughout life and enabling their participation in society according to their needs, desires, and capabilities [9]. The proposition of the theory and the concept contributes, with models that compose it, in defining a self-supporting demand. Above-mentioned requisites must be met; therefore, specific actions are required to live and maintain health [10]. However, actions have to be known to the individual as well as be within the capabilities of the person [10]. In addition, the realisation of various self-supporting activities requires a certain level of self-supporting ability [10].

In summary, self-supporting ability is the ability of older adults to give full play to their existing advantages and potentials and meet the needs of all aspects of the old-age care process, including mainly economic self-reliance, life self-care ability, and health self-maintenance ability [11]. Economic self-reliance includes receiving a stipend or other salary that meets the essential needs of life and independently controlling that income. Self-care ability includes basic activities of daily living (ADLs), such as dressing and eating, and advanced self-care abilities, such as shopping and travelling alone. Health self-maintenance ability includes the ability to maintain physical and psychological health and actively participate in societal activities [12]. Self-support is a type of pension model that conforms to the “active ageing” advocated by the World Health Organization (WHO); it can not only realize the sense of self-worth of rural empty-nesters and improve the quality of their lives but also reduce the pension burden on society and family and assist in solving their current problems with pensions [12].

A person’s sense of coherence (SOC) is considered an important factor in maintaining the individual’s position on the health-ease/dis-ease continuum and in the possible movement toward the healthy end of the continuum [13]. SOC is defined as an individual capacity or inner resource to understand the overall situation, manage inevitable stress in life, and effectively use stress resistance resources (i.e., knowledge, religion, and coping strategy) to maintain health outcomes [14]. Individuals with a strong SOC perceive their lives as more comprehensible, manageable, and meaningful, and they successfully adapt to stressful situations [14]. Thus, three components that constitute the SOC concept can be outlined: comprehensibility, manageability, and meaningfulness. SOC tends to increase with age—that is, older people tend to have a stronger SOC than younger people [15]. A strong SOC has been found to contribute to subjective well-being and quality of life in older people and to be a predictor for life satisfaction in both younger and older people [16,17]. SOC is an important determinant of self-management behaviors among elderly patients with coronary heart disease [18]. SOC is also an important determinant of perceived good health; healthy older people are more likely to report a stronger SOC than unhealthy older people [19]. SOC is also considered as a major life orientation that focuses on problem solving [20]. Since SOC is about resources for health and problem-solving, it is conceptually related to self-supporting ability, which has been shown in a study among rural older adults in China [11]. SOC is also a significant predictor for self-care in older patients at risk for undernutrition [10]. Based on the present study, considering that SOC and self-supporting ability both focus on the person’ s resources or potentiality for health, these two concepts can be investigated as separate concepts or in relation to each other. In addition, self-supporting ability among rural older adults is also affected by demographic characteristics (i.e., age, education attainment, marital status, and economic status) [20,21,22].

Most studies pointed out that the overall self-supporting ability of elderly people in rural China is weak and have shown that the causes of the differences among older adults are important [11,20,22]. In addition, they show that the ability of the elderly in northwestern rural areas is significantly lower than that of the elderly in developed areas in the east due to economic levels and medical conditions [20,22]. However, these researchers have not answered the question of whether “whole” groups have qualitative differences. In addition, the existing methods for describing the current state of the self-supporting ability of older people are primarily traditional composition ratios and means. Few studies have focused on the self-supporting ability of rural empty-nesters, and research on the protective factors of self-supporting ability is obviously insufficient.

The answer mode for different topics may vary even if the scores of two individuals in a group are the same. Therefore, it is necessary to explore the heterogeneous group differences in the self-supporting ability of empty-nesters in rural areas, which can guide the development of targeted interventions.

Moreover, data-driven statistical methods, such as K-means clustering is capable of addressing such issues, the major limitation being the arbitrary choice of cluster criteria [23,24]. LPA allows researchers to identify classes or clusters of participants (latent profiles) on the basis of their responses to continuous manifest variables [25]. This type of analysis relies on probability to estimate the likelihood that each participant belongs to a particular category [25], which can provide a more rational method for categorizing the self-supporting ability of rural empty-nesters. It not only creates differences for the largest and smallest in the classifications but also examines the accuracy and effectiveness of classification using objective indexes. Furthermore, it allows us to look at the research objects from the perspective of individuals more objectively and reveal the essence of their problems [26]. In addition, even though variable-centered approaches that test for interactions among variables may be used for this purpose as well, latent profile analysis takes measurement errors into account, which is the additional advantage of LPA.

Overall, it makes sense to conduct research on latent profile analysis based on different self-supporting ability components because self-care theory and active ageing perspectives advocate that several components of self-supporting ability—life self-care ability, economic self-reliance ability, and health self-maintenance ability, most specifically, health self-maintenance ability—include physical health self-maintenance ability, psychosocial health self-maintenance ability, and social self-adaptation ability, which have the possibility to form varying types of latent profiles. On the basis of the analysis above, to bridge gap, the latent profile analysis of self-supporting ability among rural empty-nesters in northwestern China is warranted.

**Figure 1:**
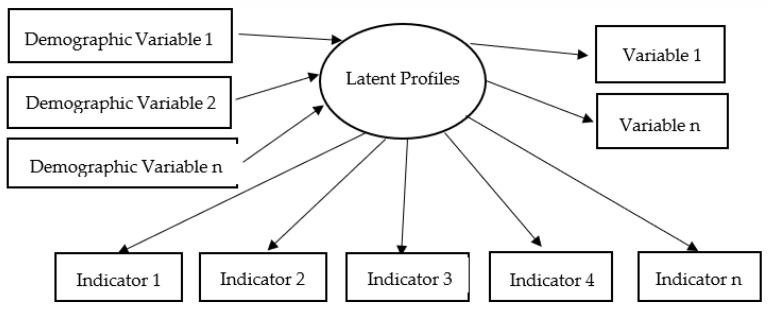
Framework diagram for the purpose of current study.

In line with the above gaps and the existing literature in this field, the present study will address the following research questions: (1) By using LPA, how many and which profiles are there in the data regarding the self-supporting ability of rural empty-nesters in northwestern China? (2) What is the size of each profile? (3) Do demographic variables and psychological factors such as a sense of coherence predict potential profile membership? Figure 1 illustrates the framework of this study.

## 2. Materials and Methods

### 2.1. Participants

This was a cross-sectional study and was approved by the Institutional Review Board (IRB) and Ethics Committee of Jiangsu University in Jiangsu, China (protocol number: 20210820). Using a convenience sampling method, 1066 eligible older adult subjects were recruited from 40 townships in Lianhua Town, Qinan County, Gansu Province (*N*_1_ = 25 townships) and Shitou Town, Luochuan County, Shanxi Province (*N*_2_ = 15 townships) in China from September 2021 to February 2022.

Eligibility criteria were as follows: (1) age ≥ 60 years; (2) no children or not living with any of their children; (3) physically and mentally competent in interviews; and (4) willing to participate in the study and to provide signed informed consent forms.

Empty-nesters were excluded from the study if they had cognitive disorders or serious diseases, such as Parkinson’s disease, Alzheimer’s disease, severe hearing impairment, acute stage of any disease, and end-stage cancer or mental disorders, such as schizophrenia, depression, and those participating in the pension institution.

### 2.2. Data Collection

Eight trained research assistants (RAs) were responsible for data collection. After obtaining the oral informed consent of potential participants, the RAs distributed the questionnaires to them and instructed them on how to complete the instruments during the door-to-door field survey. All participants who completed the questionnaire were offered ten eggs as a token of appreciation for their participation. In addition, participants were all empty-nesters who have children but do not live with them.

After collection of the questionnaires, we checked and eliminated the questionnaires that had incomplete information. The questionnaire was considered invalid if more than 15% of the items were not answered. In total, 1200 questionnaires were distributed, and 1066 valid questionnaires were included in this study, with a response rate of 88.8%. Furthermore, during the data collection process, the researchers took into account the possibility of missing values. We needed to determine the missing value pattern (e.g., MNAR or MCAR) before deciding which missing value handling method (e.g., full information, multiple imputation, or listwise deletion) to use. There were 1066 data with no missing values in the present study. We did not determine the sample size before the initiation of the project, as there was no specific hypothesis. However, a minimum sample size of 1000 subjects is recommended in studies applying the LPA method [27]. Thus, we assumed that the sample size was adequate for the purpose of the study.

### 2.3. Instruments

#### 2.3.1. The General Information Questionnaire

A self-reported demographic questionnaire was used to collect the characteristics of empty-nesters in rural areas, including age (60–69/70–79/≥80), gender (Male/Female), education level (Junior high school and below/High school or technical secondary school/Junior college or Bachelor’s degree or above), monthly income (<1000/1000–2999/≥3000), marital status (Married/Single(unmarried/divorced/widowed)), relationship with children (Good/Average/Bad), how often their children visited ((Many times per week/Once per week)/(1–2 times per month/Once per 6 months/Once per more than 6 months)/(Once per more than 12 months/Irregular visits/Never visit)), how often their children contacted them ((Many times per week/Once per week)/(1–2 times per month/Once per 6 months/Once per more than 6 months)/(Once per more than 12 months/Irregular contacts/Never contact)), relationship with neighbours (Good/Average/Bad), whether they smoked (Yes/No), whether they drank alcohol (Yes/No), the frequency of physical exercise (No/1–2 times per week/3–5 times per week/≥6 times per week), whether they had regular physical examinations (Yes/No), medical insurance (Others (no/commercial medical insurance)/Employee basic medical insurance/Basic medical insurance for urban and rural residents), and the number of chronic diseases (0/1/≥2).

#### 2.3.2. Self-Supporting Ability

The Self-supporting Ability Scale was developed by scholars from the School of Nursing, Fujian University of Traditional Chinese Medicine in China. The scale consists of seven items on economic self-supporting ability, thirteen on self-care ability, and twenty-five on health self-maintenance ability [12]. Furthermore, health self-maintenance ability includes ten items for physical health self-maintenance ability, eight items for psychological health self-maintenance ability, and seven items for social self-adaptation ability. Forty-five items were designed in the form of a 5-point Likert-type scale ranging from 1 to 5, with 1 meaning *totally nonconforming* and 5 *totally conforming.* A higher total score indicated higher levels of self-supporting ability.

#### 2.3.3. Sense of Coherence

The 13-item SOC scale developed by Antonovsky [28] can be used to evaluate individuals’ SOC. The scale consists of 13 items and includes the three dimensions of comprehensibility (five items), manageability (four items), and meaningfulness (four items). Each item was rated on a 7-point Likert scale ranging from 1 (*never*) to 7 (*always*). The total score ranges from 13 to 91, with higher scores suggesting higher levels of SOC. A score of 13 to 63 points indicates a low level of SOC, 64 to 79 indicates a moderate SOC level, and 80 to 91 indicates a high SOC level. The Chinese version of the SOC-13 was revised by Bao [29], has been used with older individuals in rural areas, and has proven to be reliable and valid [11].

### 2.4. Data Analytic Strategy

IBM SPSS 26.0 (IBM, Armonk, NY, USA) and Mplus 8.3 (Muthen & Muthen, Los Angeles, CA, USA) were used to analyse the data. Means and standard deviations were reported to describe the continuous variables, while frequencies and percentages were used to describe categorical variables.

First, different types of CFA models were fitted to the Self-Supporting Ability Scale to determine whether the scale had satisfactory psychometric properties (e.g., omega reliability, discriminant validity (average variance extracted and correlation coefficient among subscales), convergent validity, and best model fit indices). A good scale fit was indicated when *X^2^/df* was 1 to 3, CFI > 0.9, and RMSEA < 0.05.

**Figure 2:**
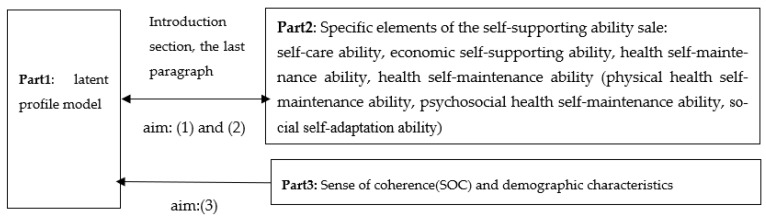
Specific framework diagram of current study.

Second, we need to specify the mean, standard deviation, skewness, and kurtosis, detect potential outliers of the self-supporting ability scale, and describe the normality distribution of each variable in order to determine which estimator (ML or MLR) to use in the latent profile analysis. When the skewness and kurtosis (absolute values) are less than 2 and 7, respectively, the variables are considered to be normally distributed, and ML can be used [30]. Then, to ensure convergence on the global maximum instead of obtaining a local solution, the multiple starting value method should be used. With this method, the number of random starts was set to 7000 and the final stage of optimization to 200 to estimate the parameters in growth mixture models [31]. Next, two constraints (local independence assumption and uncorrelated residuals) were imposed on latent profile analysis solutions to reduce the number of parameters to be estimated so as to avoid unstable solutions. Furthermore, the LPA models were fit in a stepwise manner, starting by estimating a one-prolife solution and then successively adding profile classes until the model failed to converge or improper solutions, indicating that a more parsimonious model might be necessary. Finally, Figure 2 depicts the specific framework of the study.

We performed LPA using Mplus to identify person-centred patterns of self-supporting ability. The best latent classes were finalized when the test values of Akaike’s information criterion (AIC), Bayesian information criterion (BIC), and sample-size-adjusted BIC (aBIC) reached a relative minimum [32]. The bootstrap likelihood ratio test (BLRT) and Lo–Mendell–Rubin (LMR) test were performed to compare the differential distribution of the log likelihood ratio between nested models, and statistical significance (*p* < 0.05) indicated that the K-class model was better than the K-1 model [32]. Entropy, varying between 0 and 1, implied a more accurate classification when the value was close to 1. Entropy above 0.80 indicates that 90% of individuals were precisely classified [32].

Third, the predictors of class membership were then explored. The control variables independently predicting subgroup membership were sorted by conducting chi-square analyses, Fisher’s exact tests, and one-way analysis of variance (ANOVA). Then, multivariate logistic regression analysis was used to examine the predictor best distinguishing the classes. These analyses were performed using SPSS 26.0. A *p-value* < 0.05 was regarded as statistically significant.

## 3. Results

### 3.1. Characteristics of the Study Population

The sociodemographic characteristics of the participants are presented in Table 1.

### 3.2. Model Fit Indicators for Different CFA Models of the Self-Supporting Ability Scale

We fitted a one-factor model, a two-factor model, a three-factor model, and bi-factor model to the self-supporting ability scale (Table 2). The model results showed that the three-factor model had the best model fit indicators compared with the other models; therefore, the scale had discriminant validity among the dimensions of the scale in the study, and the three-factor model had a more realistic scale structure.

### 3.3. Specific Evaluation Indicators for the Self-Supporting Ability Scale

According to Table 3, all CR values were above 0.70, and all AVEs were above 0.50; thus, the scale had good reliability. At the same time, the correlation coefficients among the dimensions were all smaller than the square root of AVE, so the dimensions had discriminant validity. In Table 3, A stands for self-care ability, B for economic self-supporting ability, and C for health self-maintenance ability.

The skewness of the three dimensions in this study ranged from −0.987 to −0.317, and the kurtosis ranged from −0.845 to −0.099, which can be considered to be in line with a normal distribution; therefore, ML analysis was used.

### 3.4. Fit Statistics for Latent Profiles: Models 1–5

Models with one to five classes were analysed (Table 4). With the increasing number of classes in the models, the values of Log(L), AIC, BIC, and BIC decreased. The LMR values of the four-and five-class models were not significant (*p* > 0.05), so the four- and five-class models were unsuitable. The two- and three-class models showed similar model fits in terms of entropy, BIC, AIC, and BLRT. However, based on the convergence of statistical evidence and the substantive interpretation of the model, the three-class model was used to best describe the various self-supporting abilities of rural empty-nesters in the study. As shown in Table 5, the average probabilities of empty-nesters belonging in each class were 99.3%, 97.6%, and 99.1%, respectively, which suggests good discriminability and accuracy of the results with the three-class model in the test sample.

**Figure 3:**
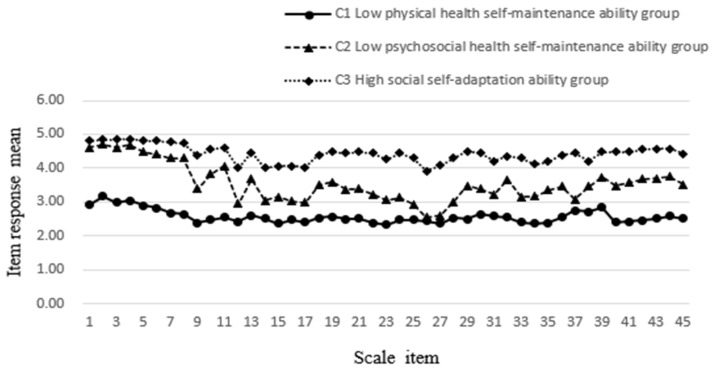
Distribution of the mean self-supporting ability scores for each of the items by subgroup.

Table 6 and Figure 3 show the pattern of the three-class solution of self-supporting ability. These three trajectories were named “low physical health self-maintenance ability” (Class 1, *n* = 219; 20.5% of the sample), “low psychological health self-maintenance ability” (Class 2, *n* = 335; 31.4% of the sample), and “high social self-adaptation ability” (Class 3, *n* = 512; 48.0% of the sample). Class 1 had the lowest levels of self-supporting ability, and the level of physical health self-maintenance was lower when compared within the group. The lowest score was for item 23, “I focus on maintaining good exercise habits (not exercising immediately after meals, hydrating properly after exercise, etc.)”, whereas Class 3 had the highest degree of self-supporting ability and a higher level of social self-adaptation when compared within the group. Item 43, “I communicate regularly with my friends and family”, and item 44, “I have adapted to my current living environment (pace of life, weather, etc.)”, had the highest scores. Class 2 was in the middle in terms of the means of the self-supporting ability scale outcome variables, and the level of mental health self-maintenance was lower when compared within the group, with the lowest score for item 24: “I accept the changes (physical, social status, etc.) that come with old age”.

### 3.5. Three Classes of Rural Empty-Nesters’ Self-Supporting Ability Scores

The total score for the self-supporting ability of rural empty-nesters was 169.29 ± 35.20. The group with low physical health self-maintenance ability scored 124.89 ± 26.73, the group with low psychological health self-maintenance ability scored 164.12 ± 24.59, and the group with high social self-adaptation ability scored 191.65 ± 22.77. Statistically significant differences were found in the scores when comparing the three classes (*F* = 594.810, *p* < 0.001).

### 3.6. Single-Factor Analysis of the Latent Classes of Self-Supporting Ability among Rural Empty-Nesters

The demographic characteristics of the three classes are shown in Table 7. Among the control variables, gender, marital status, whether they smoked, and whether they had regular physical examinations showed no significant difference across the three groups, whereas the subgroups differed significantly in the aspects of age, education level, monthly income, relationship with children, how often their children visit, how often their children contact them, relationship with neighbours, whether they drank alcohol, frequency of physical exercise, medical insurance, and the number of chronic diseases.

Table 8 presents the results of the class comparisons of the SOC and each dimension. Statistically significant differences were observed among the three classes. The average SOC was higher in the “high social self-adaptation ability” group (Class 3) than in the other classes.

### 3.7. The Influence of Characteristics on the Latent Classes of Self-Supporting Ability among Empty-Nesters in Rural Areas

Before the analysis, we treated the classes of older empty-nest adults’ self-supporting ability as the dependent variable, set “high social self-adaptation ability” (C_3_) as the reference class, treated the indicators with statistically significant differences in univariate analysis as the independent variables, and transformed the unordered multi-categorical independent variables among them into dummy variables for multinomial logistic regression analysis (Table 9). The results show that a higher level of SOC, a good relationship with neighbours, and one chronic disease were protective factors in C_1_ (OR = 0.964, 0.303, and 0.392, respectively, all *p* < 0.05). Age ≥ 80 and monthly income (RMB) < 1000 were independent risk factors in C_1_ (OR = 4.931 and 5.558, respectively, all *p* < 0.05). A higher level of SOC and many times a week or once per a week of children visiting and contacting them were protective factors in C_2_ (OR = 0.956, 0.554, and 0.400, respectively, all *p* < 0.05). (Table 10).

## 4. Discussion

This study aimed to use the multi-faceted self-supporting ability factors to identify self-supporting ability profiles of rural empty-nest elderly using LPA and examined demographic characteristics, SOC associated with self-supporting ability profiles. It illustrated that rural empty-nesters in northwestern China can be classified into three distinct self-supporting ability profiles: “low physical health self-maintenance ability” class, “low psychological health self-maintenance ability” class, and “high social self-adaptation ability” class.

### 4.1. The Heterogeneity of Self-Supporting Ability among Rural Empty-Nesters

The “low physical health self-maintenance ability” class (C_1_) comprised 20.5% of the participants. The score of self-supporting ability in this class was 124.89 ± 26.73, which was the lowest score among the three classes and was lower than the overall self-supporting ability level. Within-class comparisons revealed that older adults scored lower on the physical health self-maintenance dimension; they were less aware of their health care and lacked health-promoting lifestyles, such as regular exercise and good dietary behaviour. Health-promoting lifestyles refer to the spontaneous and multifaceted perceptions and behavior adopted by an individual to maintain his or her health promotion status and to achieve self-satisfaction and self-realization [33]. Research has shown that health-promoting lifestyles play a positive role in reducing depression and improving the quality of life among older adults [34], which has become an important topic in the field of health management. It is suggested that rural community workers pay more attention to the care of empty-nesters in this class, which can help empty-nesters make healthy choices, build pension reserves in advance, and improve their self-care and health maintenance. In addition, we should create an atmosphere of peer support and communication, allow older adults to help each other, and provide support in daily life, society, and emotion to enhance the behaviour of self-health management.

The “low psychological health self-maintenance ability” class (C_2_) comprised 31.4% of the participants. The self-supporting ability score in this class was 164.12 ± 24.59, and the level of mental health self-maintenance was lower than that within the group. These older empty-nest adults had more negative feelings and experiences of the psychological and social changes that accompany ageing. The proportion of older adults in this group with junior high school education and less and with never physical exercise status was 89.6% and 55.8%, respectively. Older people with a low level of education have limited access to information and tend to adopt negative coping methods, such as avoidance and procrastination in the face of psychological problems. This makes them unable to fully understand the resources and information around them in old age and increases their fear and insecurity about “empty nesting” [11], leading to limitations in their perception of autonomy. Due to the decline of physiological mechanisms and the change in social roles, older adults gradually develop greater spiritual needs. The underlying mechanisms might be attributed to three aspects—including physiological pathways, such as high level of stress-related C-reactive protein and cortisol levels in urine; a psychosocial pathway, such as self-efficacy; and even a behavioural pathway, such as physical activity [35]. Physical exercise is an important way for them to enrich their daily lives and support their values [36]. It is recommended that rural community workers strengthen the mental health management and services of this group in a planned, organized, and purposeful manner according to their differences in literacy and social participation to enhance their mental health self-maintenance ability and improve attitudes towards ageing.

The “high social self-adaptation ability” class (C_3_) included 48.0% of the participants. The self-supporting ability score in this group was 191.65 ± 22.77, which was the highest score among the three classes and was higher than the overall self-supporting ability level. This group had higher scores on the social self-adaptation dimension for within-group comparisons, indicating that they had a high ability to realize self-support through their potential and could actively adapt to the empty-nest life. Older individuals in this group who had a good relationship with their children (58.7%) and who received regular physical examinations (51.0%) accounted for a larger proportion. The family is an important part of the social support system. Good intergenerational relationships mean that offspring can provide their parents with resources that can effectively increase older adults’ ability to cope with empty-nest life, such as advice on solving problems and economic and emotional support, which can make older adults feel that they are surrounded by love and may stimulate their confidence in supporting themselves. Mental, physical, and social health is interdependent, and poor physical health affects mental well-being, while mental illness increases mortality and morbidity [37]. In medical terms, there are five stages of the disease: the vulnerable stage, the pre-clinical stage, the clinical stage, the recovery stage, and the death stage, the first and second of which are not yet obvious to the patient. The physical examination is the screening of the patient’s organs and tissues to understand the patient’s health status, to detect diseases that do not show clinical symptoms but are caused by risk factors, and to curb the occurrence and development of diseases through a series of methods and means, such as changing bad habits and diet [38]. At the same time, physical examination can also detect malignant tumours, blood disorders, and diseases of various organs, enabling early detection and treatment while improving the quality of life and prolonging the survival time of patients and avoiding the irreversible effects and damage caused by late diagnosis of diseases after the appearance of obvious symptoms [38]. Overall, regular physical examination indicates that empty-nesters have a good awareness of health promotion, which can enhance their sense of control in terms of life and psychology. We may guide people in this group and help them play a key role for other empty-nest adults and further explore targeted intervention strategies on how to improve the self-supporting ability of empty-nesters. The local government may call for senior volunteers (high social self-adaptation ability) to provide peer counselling on physical and mental health problems.

### 4.2. The Demographic Characteristics and SOC among Different Self-Supporting Ability Classes of Rural Empty-Nest Elderly

Further study showed that a greater sense of coherence (SOC), age ≥ 80, monthly income (RMB) < 1000, a good relationship with neighbours, and one type of chronic disease were significantly associated with “low physical health self-maintenance ability” when compared with “high social self-adaptation ability” (*p* < 0.05). In addition, a greater SOC, a high frequency of their children visiting and contacting them were more significantly related to “low psychological health self-maintenance ability” than to “high social self-adaptation ability” (*p* < 0.05).

We found that SOC was a factor that positively influences the way older empty-nest adults in rural areas to adapt to self-supporting conditions, indicating that the higher the level of SOC is, the lower the risk that these adults will enter the groups with low physical health self-maintenance ability (C_1_) and low psychological health self-maintenance ability (C_2_) (OR = 0.964 and 0.956, respectively). Antonovsky indicates that SOC can induce patterns of responses to situations that stimulate the brain to send messages to activate appropriate bodily resources and cause one to realize the need to cope both instrumentally and emotionally [15]. Therefore, empty-nesters with a strong SOC can understand that the empty-nest stress from the internal and external environments is structured and predictable and to use available resources to manage stress and promote health [15]. They are, thus, motivated to improve their physical and psychological health self-maintenance ability. Therefore, the higher the SOC score is, the more that rural empty-nesters can utilize their advantages and potential and meet their needs through self-support.

Age ≥80 and monthly income (RMB) <1000 were risk factors in class 1 (OR = 4.931 and 5.558, respectively). Older adults are unable or have difficulty maintaining a healthy lifestyle due to declines in physical function (e.g., physical dysfunction, cognitive impairment, etc.). Cognitive function mirrored individual’s mental status, which interacts with physical dysfunction among aged people. The reaction of external stimuli was reduced when a series of somatic diseases emerged; afterwards, the cognitive damage would reversely influence the body function [35]. Connolly et al. pointed out that cognitive impairment significantly decreased the ability of health promotion because of degenerative language and mobility [39]. In addition, previous research indicated the relationship of socioeconomic status to health [40]. Some also showed the effect of income on health after controlling the factor of education [41]. Economic status can influence the frequency and intensity of health behaviours adopted by older adults. Low demand for and ability to achieve health services among economically disadvantaged older people results in low motivation to engage in health behaviours [42], both of which affect the physical health self-maintenance ability among empty-nesters in rural areas.

Maintaining good neighbourhood relationships was a protective factor in Class 1 (OR = 0.303). This suggests that informal social networks with neighbours could promote the development of physical health self-maintenance ability in empty-nesters. There are several possible reasons for this result. First, having a social network with neighbours may help to relieve rural empty-nesters’ loneliness. According to activity theory, human beings require a sense of achievement and belonging within the social network [35]. Rural empty-nest elderly are often socially isolated and lonely, and so having a connection with neighbours may give them a sense of belonging to the rural community. Second, the social escort convoy theory model suggests that individuals are surrounded by supporters in their social networks throughout their lives, and these social relationships have an escort function for the individual’s health [43].Good neighbourhood relationships may increase opportunities for social support. A previous study reported that receiving informal social support was inversely related to the degree of impairment in the physical and mental health of older adults [44]. Third, rich neighbourhood relationships could positively contribute to residents’ health through providing access to resources, including expert advice and problem-solving resources [45]. These resources may help older adults overcome the adverse effects [46] of empty nest status on their physical health. Asians, including Chinses people, traditionally value their relationships with others, such as with neighbours. Thus, maintaining good neighbourhood relationships might be necessary to improve rural empty-nesters’ confidence in self-support. However, these explanations remain speculative, and further research is needed to examine the mechanisms underlying this result.

The present study found that having one chronic disease was an independent protective factor in Class 1 (OR = 0.392), indicating that individuals with this characteristic were less likely to be assigned to Class 1. The possible reason is that clinical treatment and care for a single chronic disease are reliable, and reliance on medication regimens can provide better control and relief of symptoms [47]. Individuals with one chronic disease are less likely to assess their health status correctly; their self-assessment of their health status does not differ from that of people without a chronic disease [48]. People with one chronic disease may experience positive changes as they gradually adapt to their disease, such as the learning of self-management strategies for chronic illness [47]. Chronic disease self-management (CDSM) involves daily strategies an individual uses to control their diseases [49], such as exercise, diet, health checkups, and medication. Interventions targeting self-management are effective and affordable. Efficient self-management of older patients with chronic diseases may improve disease prognosis and life quality, which may mitigate the negative effects of having a chronic condition on self-support.

Children visiting and contacting their parents with many times per week or once per week were related to older adults who were less likely to be assigned to C_2_ (OR = 0.554 and 0.400, respectively), showing that a higher level of intergenerational support can alleviate the psychological stress of older empty-nest adults. Intergenerational solidarity theory suggests that affection and love between parents and adult children may be one of the most important motivations for children to support their parents, and there is a positive relationship between high levels of affection and intergenerational support for older adults [21]. A previous study showed that filial piety was not only an important form of support for elderly individuals’ psychological well-being but also an important resource for their emotional needs [50]. High-level intergenerational support can enhance the positive psychological experiences of older adults, promote their mental health, and help them better adapt to empty-nest life, thereby improving their self-support awareness.

## 5. Conclusions

The difference between this study and previous studies is that we carried out LPA among rural empty-nesters as subgroups rather than as a homogeneous whole. We divided the subjects into three profiles. These profiles are “low physical health self-maintenance ability” class, “low psychological health self-maintenance ability” class, and “high social self-adaptation ability” class. In the future, applying and studying tailored interventions for these sub-profiles will expand on our current research and further contribute to the literature. We also identified sense of coherence (SOC), age, monthly income (RMB), the relationship with neighbours, the frequency of their children visiting and contacting them, and the number of chronic diseases associated with profile membership. These characteristics can provide rural community staff with crucial actionable intervention points to minimize psychosocial risks and improve the self-supporting ability of empty-nesters living in rural areas.

## 6. Limitations

There are two limitations that must be discussed. First, the sample size is large; however, the empty-nesters come from two provinces in China and are mainly in the northwestern Chinese provinces of Gansu and Shanxi, which are economically and medically disadvantaged compared with the coastal provinces of southeast China. Therefore, the results are not generalizable. Future research can be conducted to compare different countries or different regions of China to find similarities and differences in the latent classes and predictors of rural-empty nest older adults’ self-supporting ability. Second, this study could not show the dynamic patterns of self-supporting ability from a longitudinal perspective. Future research can compare longitudinal changes in self-supporting ability among rural empty-nesters.

## Figures and Tables

**Table 1:** General characteristics (N = 1066).

Variable		N (%)
Gender	Male	574 (53.8%)
	Female	492 (46.2%)
Age	60–69	468 (43.9%)
	70–79	459 (43.1%)
	≥80	139 (13.0%)
Education level	Junior high school and below	959 (90.0%)
	High school or technical secondary school or Junior college or Bachelor’s degree or above	107 (10.0%)
Monthly income (RMB)	<1000	858 (80.5%)
	1000–2999	146 (13.7%)
	≥3000	62 (5.8%)
Marital status	Married	785 (73.6%)
	Single (unmarried/divorced/widowed)	281 (26.4%)
Relationship with children	Good	537 (50.4%)
	Average	486 (45.6%)
	Bad	43 (4.0%)
How often the children visit them	Many times per week/Once per week	238 (22.3%)
	1–2 times per month/Once per 6 months/Once per more than 6 months	678 (63.6%)
	Once per more than 12 months/Irregular visits/Never visit	150 (14.1%)
How often the children contact them	Many times per week/Once per week	393 (36.9%)
	1–2 times per month/Once per 6 months/Once per more than 6 months	616 (57.8%)
	Once per more than 12 months/Irregular contacts/Never contact	57 (5.3%)
Relationship with neighbours	Good	399 (37.4%)
	Average	608 (57.0%)
	Bad	59 (5.5%)
Whether they smoked	Yes	342 (32.1%)
	No	724 (67.9%)
Whether they drank alcohol	Yes	347 (32.6%)
	No	719 (67.4%)
The frequency of physical exercise	No	560 (52.5%)
	1–2 times per week	312 (29.3%)
	3–5 times per week	110 (10.3%)
	≥6 times per week	84 (7.9%)
Whether they have regular physical examinations	Yes	473 (44.4%)
	No	593 (55.6%)
Medical insurance	Others (no/commercial medical insurance)	3.9 (4.2%)
	Employee basic medical insurance	78 (7.3%)
	Basic medical insurance for urban and rural residents	946 (88.7%)
The number of chronic diseases	0	261 (24.5%)
	1	410 (38.5%)
	≥2	395 (37.1%)

**Table 2:** Model fit indicators for different CFA models of the Self-Supporting Ability Scale.

Model	*X^2^*	*df*	*X^2^/df*	RMSEA	CFI	TLI	SRMR
One-factor model	241.882	12	20.157	0.134	0.951	0.915	0.033
Two-factor model	139.252	11	12.659	0.105	0.973	0.948	0.027
Three-factor model	86.170	9	9.574	0.090	0.984	0.962	0.022
Bi-factor model	236.771	8	29.596	0.164	0.952	0.873	0.336

**Table 3:** Specific evaluation indicators for the Self-Supporting Ability Scale.

	(M (SD))	Kurtosis	Skewness	95% CI of Omega	CR	AVE	A	B	C
A	53.33 (11.21)	−0.099	−0.987	0.005–0.019	0.800	0.668	0.817		
B	24.83 (6.90)	−0.635	−0.317	0.010–0.027	0.746	0.595	0.614	0.771	
C	91.12 (20.95)	−0.845	−0.348	0.008–0.014	0.877	0.704	0.696	0.708	0.839

**Table 4:** Fit statistics for latent profiles: Models 1–5.

Model	K	Log(L)	AIC	BIC	aBIC	Entropy	LMR	BLRT	Class Probability
1	90	−75,202.843	150,585.686	151,033.052	150,747.196	-	-	-	-
2	136	−66,010.938	132,293.877	132,969.896	132,537.936	0.981	<0.0001	<0.0001	0.331/0.669
3	182	−63,349.548	127,063.096	127,967.769	127,389.705	0.970	0.0048	<0.0001	0.205/0.314/0.480
4	228	−62,207.046	124,870.092	126,003.419	125,279.250	0.959	0.4924	<0.0001	0.131/0.176/0.335/0.358
5	274	−61,281.623	123,111.245	124,473.225	123,602.953	0.969	0.2233	<0.0001	0.127/0.139/0.243/0.115/0.377

**Table 5:** The probability of latent profile analysis.

	C_1_	C_2_	C_3_
C_1_	0.993	0.007	0.000
C_2_	0.004	0.976	0.020
C_3_	0.000	0.009	0.991

**Table 6:** The 45-item response mean of the self-supporting ability scale of the three classes.

Dimension		Items	C_1_	C_2_	C_3_
Self-care ability	Basic self-care ability	1	2.92	4.61	4.81
		2	3.17	4.70	4.85
		3	2.98	4.61	4.85
		4	3.04	4.68	4.87
		5	2.90	4.48	4.81
		6	2.81	4.40	4.81
		7	2.67	4.29	4.76
		8	2.64	4.29	4.73
	Advanced self-care ability	9	2.38	3.39	4.37
		10	2.47	3.82	4.56
		11	2.54	4.07	4.61
		12	2.41	2.95	4.00
		13	2.59	3.67	4.46
Economic self-supporting ability	Income self-sufficiency ability	14	2.51	3.04	4.00
		15	2.37	3.15	4.05
		16	2.47	3.02	4.05
		17	2.39	2.99	4.02
	Income self-determination ability	18	2.53	3.49	4.39
		19	2.57	3.59	4.47
		20	2.49	3.36	4.45
Health self-maintenance ability	Physical health self-maintenance ability	21	2.51	3.38	4.48
		22	2.38	3.21	4.45
		23	2.32	3.06	4.26
		24	2.47	3.14	4.44
		25	2.48	2.93	4.30
		26	2.44	2.55	3.92
		27	2.37	2.60	4.07
		28	2.52	3.00	4.30
		29	2.50	3.48	4.49
		30	2.64	3.39	4.44
	Psychosocial health self-maintenance ability	31	2.58	3.22	4.20
		32	2.55	3.65	4.35
		33	2.40	3.15	4.30
		34	2.36	3.16	4.12
		35	2.38	3.35	4.20
		36	2.55	3.46	4.38
		37	2.74	3.07	4.45
		38	2.71	3.45	4.20
	Social self-adaptation ability	39	2.84	3.74	4.49
		40	2.41	3.48	4.48
		41	2.42	3.57	4.48
		42	2.45	3.68	4.55
		43	2.51	3.69	4.57
		44	2.59	3.76	4.57
		45	2.51	3.52	4.42

These 45 items are displayed by dimension. Items 1–13 belong to self-care ability, items 14–20 belong to economic self-supporting ability, and items 21–45 belong to health self-maintenance ability. Health self-maintenance ability includes 21–30 items for physical health self-maintenance ability, 31–38 items for psychosocial health self-maintenance ability, and 39–45 items for social self-adaptation ability.

**Table 7:** Demographic characteristics of the three classes.

Variable		Latent Profile (N(%))			X^2^	*p*-Value
		C_1_	C_2_	C_3_		
Gender	Male	115 (20.0%)	174 (30.3%)	285 (49.7%)	1.327	0.515
	Female	104 (21.1%)	161 (32.7%)	227 (46.1%)		
Age	60–69	79 (16.9%)	153 (32.7%)	236 (50.4%)	17.213	0.002
	70–79	95 (20.7%)	150 (32.7%)	214 (46.6%)		
	≥80	45 (32.4%)	32 (23.0%)	62 (44.6%)		
Education level	Junior high school and below	207 (21.6%)	300 (31.3%)	452 (47.1%)	6.704	0.035
	High school or technical secondary school or Junior college or Bachelor’s degree or above	12 (11.2%)	35 (32.7%)	60 (56.1%)		
Monthly income (RMB)	<1000	208 (24.2%)	264 (30.8%)	386 (45.0%)	40.569	0.000
	1000–2999	8 (5.5%)	54 (37.0%)	84 (57.5%)		
	≥3000	3 (4.8%)	17 (27.4%)	42 (67.7%)		
Marital status	Married	171 (21.8%)	236 (30.1%)	378 (48.2%)	3.994	0.136
	Single (unmarried/divorced/widowed)	48 (17.1%)	99 (35.2%)	134 (47.7%)		
Relationship with children	Good	54 (10.1%)	168 (31.3%)	315 (58.7%)	85.240	0.000
	Average	151 (31.1%)	150 (30.9%)	185 (38.1%)		
	Bad	14 (32.6%)	17 (39.5%)	12 (27.9%)		
How often the children visit them	Many times per week/Once per week	17 (7.1%)	58 (24.4%)	163 (68.5%)	67.711	0.000
	1–2 times a month/Once per 6 months/Once per more than 6 months	178 (26.3%)	216 (31.9%)	284 (41.9%)		
	Once per more than 12 months/ Irregular visits/Never visit	24 (16.0%)	61 (40.7%)	65 (43.3%)		
How often the children contact them	Many times per week/Once per week	31 (7.9%)	116 (29.5%)	246 (62.6%)	89.254	0.000
	1–2 times per month/Once per 6 months/Once per more than 6 months	173 (28.1%)	189 (30.7%)	254 (41.2%)		
	Once per more than 12 months/ Irregular visits/Never visit	15 (26.3%)	30 (52.6%)	12 (21.1%)		
Relationship with neighbours	Good	37 (9.3%)	122 (30.6%)	240 (60.2%)	68.226	0.000
	Average	162 (26.6%)	187 (30.8%)	259 (42.6%)		
	Bad	20 (33.9%)	26 (44.1%)	13 (22.0%)		
Smoking	Yes	68 (19.9%)	102 (29.8%)	172 (50.3%)	1.055	0.590
	No	151 (20.9%)	233 (32.2%)	340 (17.0%)		
Drinking	Yes	91 (26.2%)	106 (30.5%)	150 (43.2%)	10.678	0.005
	No	128 (17.8%)	229 (31.8%)	362 (50.3%)		
The frequency of physical exercise	No	99 (17.7%)	187 (33.4%)	274 (48.9%)	27.442	0.000
	1–2 times per week	86 (27.6%)	101 (32.4%)	125 (40.1%)		
	3–5 times per week	24 (21.8%)	21 (19.1%)	65 (59.1%)		
	≥6 times per week	10 (11.9%)	26 (31.0%)	48 (57.1%)		
Whether they have regular physical examinations	No	123 (20.7%)	199 (33.6%)	271 (45.7%)	3.470	0.176
	Yes	96 (20.3%)	136 (28.8%)	241 (51.0%)		
Medical Insurance	Others (no/commercial medical insurance)	6 (14.3)	16 (38.1)	20 (47.6)	14.536	0.006
	Employee basic medical insurance	7 (9.0)	19 (24.4)	52 (66.7)		
	Basic medical insurance for urban and rural residents	206 (21.8)	300 (31.7)	440 (46.5)		
The number of chronic diseases	0	41 (15.7%)	88 (33.7%)	132 (50.6%)	48.053	0.000
	1	55 (13.4%)	127 (31.0%)	228 (55.6%)		
	≥2	123 (31.1%)	120 (30.4%)	152 (38.5%)		

**Table 8:** Class comparisons on the sense of coherence.

Variable	Latent Profile (M(SD))			*F*	*p*
	C_1_	C_2_	C_3_		
SOC	52.51 (5.83)	54.15 (8.51)	58.44 (10.22)	42.880	0.000
Manageability	17.44 (2.38)	18.03 (3.06)	19.07 (3.39)	24.782	0.000
Comprehension	20.15 (2.67)	20.68 (3.71)	22.39 (4.37)	34.031	0.000
Meaningfulness	14.91 (2.35)	15.44 (3.16)	16.98 (3.67)	39.652	0.000

**Table 9:** The dummy variable assignment of multivariate logistic regression.

Factor	Variable	Assignment Instructions
Sense of coherence	X1	Measured
Education level	X2	Junior high school and below = 1
		High school or technical secondary school or Junior college or Bachelor’s degree or above = 0
Medical insurance	X2	Set the dummy variable with “Others (no/commercial medical insurance)” as the reference
		Employee basic medical insurance = 1, else = 0
		Basic medical insurance for urban and rural residents = 1, else = 0
Age	X3	Set the dummy variable with ”≥80” as the reference
		60–69 = 1, else = 0
		70–79 = 1, else = 0
Monthly income (RMB)	X5	Set the dummy variable with “≥3000” as the reference
		<1000 = 1, else = 0
		1000–2999 = 1, else = 0
The relationship with children	X6	Good = 1, average = 2, bad = 3
How often the children visit them	X7	Set the dummy variable with “Once per more than 12 months/Irregular visits/Never visit“ as the reference
		Many times per week/Once per week = 1, else = 0
		1–2 times per month/Once per 6 months/Once per more than 6 months = 1, else = 0
How often the children contact them	X8	Set the dummy variable with “Once per more than 12 months/Irregular contacts/Never contact“ as the reference
		Many times per week/Once per week = 1, else = 0
		1–2 times per month/Once per 6 months/Once per more than 6 months = 1, else = 0
The relationship with neighbours	X9	Good = 1, average = 2, bad = 3
Drinking	X10	Yes = 1, No = 0
The frequency of physical exercise	X11	Set the dummy variable with “≥6 times per week” as the reference
		No = 1, else = 0
		1–2 times per week = 1, else = 0
		3–5 times per week = 1, else = 0
The number of chronic diseases	X12	Set the dummy variable with “≥2” as the reference
		0 = 1, else = 0
		1 = 1, else = 0

**Table 10:** Multinomial logistic regression of latent classes on characteristics.

Variable			B	SE	WaldX^2^	OR	95% CI	*p*-Value
C_1_ vs. C_3_ (ref)	SOC		−0.036	0.013	8.059	0.964	0.941–0.989	0.005
	Education level (ref = High school or technical secondary school or Junior college or Bachelor’s degree or above)	Junior high school and below = 1	0.565	0.315	3.209	1.759	0.948–3.264	0.073
	Medical Insurance (ref = Others (no/commercial medical insurance))	Employee Basic Medical Insurance	−0.199	0.532	0.140	0.819	0.289–2.324	0.708
		Basic Medical insurance for urban and rural residents	−0.110	0.526	0.044	0.896	0.319–2.514	0.835
	Age (ref = 60–69)	70–79	0.666	0.686	0.941	1.946	0.507–7.468	0.332
		≥80	1.596	0.219	52.864	4.931	3.207–7.581	0.000
	Monthly income (RMB) (ref = ≥3000)	<1000	1.715	0.658	6.795	5.558	1.530–20.184	0.009
		1000–2999	0.310	0.756	0.169	1.364	0.310–5.998	0.681
	The relationship with children (ref = bad)	good	−0.422	0.529	0.635	0.656	0.232–1.851	0.425
		general	0.118	0.497	0.056	1.125	0.425–2.981	0.812
	How often the children visit them (ref = Once per more than 12 months/Irregular visits/Never visit)	high	−0.174	0.434	0.161	0.840	0.359–1.966	0.688
		general	0.510	0.323	2.486	1.665	0.883–3.138	0.115
	How often the children contact them (ref = Once per more than 12 months/Irregular contacts/Never contact)	high	−0.748	0.549	1.857	0.473	0.161–1.388	0.173
		general	−0.233	0.478	0.238	0.792	0.310–2.020	0.625
	Relationships with neighbours (ref = bad)	good	−1.194	0.479	6.224	0.303	0.119–0.774	0.013
		general	−0.727	0.433	2.822	0.483	0.207–1.129	0.093
	Drinking (ref = no)	yes	−0.339	0.198	2.927	0.712	0.483–1.051	0.087
	Frequency of physical exercise (ref =≥ 6 times per week)	no	0.124	0.423	0.086	1.132	0.494–2.593	0.770
		1–2 times per week	0.727	0.426	2.911	2.069	0.898–4.768	0.088
		3–5 times per week	0.809	0.488	2.741	2.245	0.862–5.846	0.098
	Number of chronic diseases (ref =≥ 2)	0	−0.342	0.255	1.792	0.711	0.431–1.172	0.181
		1	−0.937	0.215	18.940	0.392	0.257–0.598	0.000
C_2_ vs. C_3_ (ref)	SOC		−0.045	0.009	24.004	0.956	0.939–0.973	0.000
	Education level (ref = High school or technical secondary school or Junior college or Bachelor’s degree or above)	Junior high school and below = 1	−0.345	0.318	1.175	0.708	0.379–1.322	0.278
	Medical Insurance (ref = Others (no/commercial medical insurance))	Employee basic medical insurance	−0.047	0.271	0.030	0.954	0.561–1.621	0.862
		Basic medical insurance for urban and rural residents	−0.437	0.362	1.462	0.646	0.318–1.312	0.227
	Age (ref = 60–69)	70–79	0.301	0.265	1.291	1.351	0.804–2.269	0.256
		≥80	0.279	0.259	1.154	1.321	0.795–2.197	0.283
	Monthly income (RMB) (ref =≥ 3000)	<1000	0.114	0.332	0.118	1.121	0.584–2.149	0.732
		1000–2999	0.219	0.367	0.357	1.245	0.607–2.556	0.550
	Relationship with children (ref = bad)	good	0.154	0.473	0.106	1.166	0.461–2.950	0.745
		general	−0.011	0.459	0.001	0.989	0.402–2.431	0.980
	How often the children visit them (ref = Once per more than 12 months/Irregular visits/Never visit)	high	−0.591	0.278	4.524	0.554	0.321–0.955	0.033
		general	0.020	0.233	0.008	1.020	0.646–1.612	0.931
	How often the children contact them (ref = Once per more than 12 months/Irregular contacts/Never contact)	high	−0.915	0.444	4.254	0.400	0.168–0.956	0.039
		general	−0.940	0.409	5.281	0.390	0.175–0.871	0.022
	Relationships with neighbours (ref = bad)	good	−0.663	0.411	2.596	0.515	0.230–1.154	0.107
		general	−0.723	0.391	3.414	0.485	0.225–1.045	0.065
	Drinking (ref = no)	yes	−0.088	0.169	0.273	0.916	0.657–1.275	0.601
	Frequency of physical exercise (ref =≥ 6 times per week)	no	−0.064	0.292	0.048	0.938	0.529–1.663	0.827
		1–2 times per week	0.073	0.297	0.061	1.076	0.602–1.925	0.805
		3–5 times per week	−0.576	0.368	2.448	0.562	0.273–1.157	0.118
	Number of chronic diseases (ref =≥ 2)	0	0.008	0.210	0.002	1.008	0.668–1.522	0.969
		1	−0.248	0.178	1.942	0.781	0.551–1.106	0.163

## Data Availability

The data presented in this study are available on request from the corresponding author and first author.
